# The trend of normal vaginal delivery and cesarean sections before and after implementing the health system transformation plan based on ICD‐10 in the northeast of Iran: A cross‐sectional study

**DOI:** 10.1002/hsr2.1131

**Published:** 2023-03-13

**Authors:** Masoumeh Sarbaz, Seyyedeh Fatemeh Mousavi Baigi, Fereshte Manouchehri Monazah, Nooshin Dayani, Khalil Kimiafar

**Affiliations:** ^1^ Department of Health Information Technology, School of Paramedical and Rehabilitation Sciences Mashhad University of Medical Sciences Mashhad Iran; ^2^ Student Research Committee Mashhad University of Medical Sciences Mashhad Iran; ^3^ Department of Medical Informatics, School of Medicine Mashhad University of Medical Sciences Mashhad Iran

**Keywords:** cesarean section, health system transformation plan, ICD‐10, normal vaginal delivery

## Abstract

**Background and Aims:**

Concerning the growing rate of cesarean sections (CSs) worldwide, encouraging normal vaginal deliveries (NVDs) and mitigating CS rates is a necessity. This study investigated the status of delivery in hospitals affiliated with the Mashhad University of Medical Sciences (MUMS) before and after implementing health system transformation plan (HSTP).

**Methods:**

A cross‐sectional study was conducted in 2017 in the obstetrics and gynecology ward in four MUMS teaching hospitals. Data were extracted from hospital information systems (HISs) based on the International Classification of Diseases (ICD‐10) and analyzed in SPSS VE10 software.

**Results:**

The results revealed a significant difference between the rate of NVDs and CSs before and after HSTP, such that implementing this plan in MUMS hospitals has raised the rate of NVDs by 4%. Except for the age groups of less than 15 and 36–40 years, the difference between NVD and CS was significant in different age groups before and after HSTP. The rate of NVD significantly increased within 2 months after implementing HSTP. Furthermore, the difference in the rate of previous CS before and after implementing HSTP was significant (*p* < 0.001).

**Conclusion:**

The results of this study show the positive impact of the implementation of the HSTP on CS reduction and NVD increase in the studied hospitals. Since the studied hospitals were teaching and concerning the different costs of NVD and CS between the public and private hospitals, it is recommended to study all hospitals with the obstetrics and gynecology ward to precisely assess the success of HSTP in encouraging NVD.

## 1 INTRODUCTION

Health is among the most critical pillars of social and economic progress. A central health indicator in any country is maternal health. According to World Health Organization (WHO) guidelines, nations must enhance maternal and infant health by diminishing cesarean sections (CSs) rates.^1^ In Iran, the maternal mortality rate (MMR) has dropped by 72%, that is, from 83 deaths per 100,000 live births in 1990 to 23 deaths in 2013. However, it is vital to develop programs to enhance maternal health in the country.^2^ In recent decades, the CS rate has grown immensely in middle‐ and high‐income nations worldwide.^3^, ^4^ According to the WHO report, the standard CS rate was found in only 14 countries out of 137 in 2010.^5^ According to the Iranian Ministry of Health and Medical Education (MHME), the CS rate has risen from 40.7% in 2005 to 53% in 2014.^6^ It is much higher in private hospitals and reaches 90%.^7^ According to the WHO report, the CS rate should not exceed 15% of all deliveries.^8^ However, research has reported a higher CS incidence in Iran.^9^ Despite the declining trend in MMR in Iran from 1990 to 2013, CS outcomes still account for most maternal mortality in the country.^2^ While, if conducted correctly and at the right time, CS can avoid mortality and other severe outcomes among mothers and infants, evidence suggests that CS may predispose healthy women and infants to increased mortality risk.^10^ Unnecessary CS leads to increased complications among mothers and fetuses and imposes remarkable health care costs.^11^

Many studies have shown that women who undergo CS unnecessarily are at high risk of complications and death.^12^ In addition, CS infants are more likely to develop respiratory problems, obesity, and other metabolic conditions.^13^ The CS rate exceeds its threshold, and no sufficient data supports CS benefits for both mothers and infants.^14^ A needless CS is a classic example of the gap between evidence and practice in women's health and delivery. In developing nations, maternal and infant health enhancements rely on upgrading health systems. In cases of rare resources, the increased CS trend leads to a further shortage of resources.^15^ Lack of sufficient information about CS outcomes, fear of pain, psychological stress, and short delivery times substantially contribute to undergoing CS.^16^, ^17^

Improving the quality of health care during delivery is crucial to diminishing morbidity and mortality in mothers and infants, while CS is associated with higher risks for the disease than vaginal delivery.^18^ Iranian health policymakers and decision‐makers have proposed multiple plans to reduce the CS rate in the country.^19^ Encouraging normal vaginal deliveries (NVDs) is among the MHME's top priorities, aiming to ensure the quality of maternal care.^14^

The Iranian MHME has developed a health system transformation plan (HSTP) to encourage NVD and improve maternal and infant health in public hospitals. This plan has been implemented nationwide since May 5, 2014. According to the HSTP, all hospitals must reduce CS rates by 10% per year. Other HSTP goals include population growth supported by health insurance, improving the quality of care in MHME hospitals, cutting health care costs, and updating treatment tariffs proportional to the actual value of services.^20^

In addition, the plan to promote NVD was implemented on April 5, 2014, in all MHME hospitals. Declining the CS rate to enhance maternal and neonatal health indicators was among the NVD objectives. The NVD program sought to reduce CS rates by 10% by 2015, keep privacy, provide more convenient delivery rooms to increase maternal satisfaction, motivate obstetricians and midwives with incentive tariffs for vaginal deliveries, and pay anesthesiologists for painless deliveries.^21^ Therefore, NVD was conducted freely in public hospitals. The NVD program further focused on using pharmaceutical and nonpharmacological methods to reduce pain by encouraging public agencies and service providers. Delivery preparation courses were provided for pregnant mothers to encourage the culture of pregnancy and childbirth while simultaneously supporting health care providers to promote NVD.

Although the impact of HSTP on CS reduction has been studied in some cities, including Qom^22^ and Kerman.^23^ However, no study has been conducted to investigate the effect of reducing CSs before and after the implementation of HSTP in Mashhad, the second largest city in Iran. Research into HSTP can help evaluate the overall impact of this plan on CS and improve decision‐making to develop and propose similar programs in the future.^14^ This study investigated the status of delivery in hospitals affiliated with MUMS before and after HSTP.

## 2 METHODS

### 2.1 Study design and environment

A cross‐sectional study was conducted in 2017 in four MUMS teaching hospitals having obstetrics and gynecology wards. The records of all women referred to these hospitals from May to November 2013 and 2014, that is, before and after implementing HSTP, were reviewed.

Mashhad is the largest city in Iran after Tehran, which occupies about 118,854 km^2^ of Iran. MUMS is one of the state universities of Iran and one of the best universities of medical sciences in Iran under the Ministry of Health, Treatment and Medical Education in Mashhad.

This university is considered one of the largest universities in the country with 7 vice‐chancellors, 18 health and treatment networks, 7 faculties, 28 hospitals, 16 research centers, and in general, in terms of the wide scope of providing health care services to about 5 million people.

### 2.2 Data collection

The main categories, subcategories, and codes relevant to each type of delivery and the delivery outcome, including single or multiple births, alive or dead baby deliveries, and the place of birth, were extracted according to the International Classification of Diseases, 10th revision (ICD‐10)‘s chapter allocated to childbirth.

ICD‐10 is the 10th edition of the International (Statistical) Classification of Diseases (and Related Health Problems) published by WHO. The purpose of ICD‐10 is to be able to systematically collect, compare, interpret, and analyze the information collected in an integrated manner about diseases and deaths in different countries and regions at different times.^22^

Data accessibility and extraction from patients' records and existing information systems were provided by coding patients' records in teaching hospitals by the medical coder.

Data were extracted from the hospital information system (HIS), as assisted by the Department of Health Information Management in teaching hospitals. First, the data were cleaned. In the first step, the data was screened for possible duplicates based on the national identity, medical record number, age, and date of admission. Then the cases with incomplete or wrongly registered ICD‐10 codes of delivery were omitted. Furthermore, any records with incorrect information due to incorrect coding were removed.

### 2.3 Statistical analysis

In this study, HSTP independent variables and ICD‐10 codes related to the status of delivery were considered dependent variables. The records were coded, and the data were entered and analyzed using SPSS version 10.0 software.

Frequency and percentage were used to analyze descriptive data, and mean and standard deviation were used to describe continuous variables. *χ* ^2^ and Pearson tests were used to analyze the relationship between classification variables. A *p* value of 0.05 was used to determine the level of statistical significance.

## 3 RESULTS

The number of deliveries in four MUMS teaching hospitals in Mashhad during 6 months in 2013 and 2014 was 7541 and 10,687, respectively, implying a 41.7% increase in the rate of deliveries in 2014 over 2013. Similarly, the number of NVDs in 2013 and 2014 was 4327 and 6562, and the corresponding number for CS deliveries was 3214 and 4152, respectively. The ratio of NVDs to all deliveries in 2013 and 2014 was 57.4% and 61.4%, respectively. The mean age of the women attending the study was 28 years, ranging from 10 to 65. Of all mothers, 11.7% were under 20 years old, 76.7% were 20–35 years old, and 11.6% were over 36 years old (Table 1).

**Table 1:** Relationship between mothers' age groups and type of delivery before and after implementing HSTP.

	Before HSTP		After HSTP		Frequency total	Percentage total
	Frequency	Percentage	Frequency	Percentage		
<15	27	0.4	30	0.3	57	0.3
16–20	903	12.0	1179	11.0	2082	11.4
21–25	2023	26.8	2762	25.8	4785	26.3
26–30	2228	29.5	3202	30.0	5430	29.8
31–35	1486	19.7	2277	21.3	3763	20.6
36–40	682	9.0	1005	9.4	1687	9.3
>40	160	2.1	213	2.0	373	2.0
0	32	0.4	19	0.2	51	0.3

Abbreviation: HSTP, health system transformation plan.

The nonparametric *χ* ^2^ test was used to test the difference between the number of NVDs and CS deliveries before and after implementing HSTP. With a 99% probability, the difference between the number of NVDs and CS deliveries following the implementing HSTP was significant (*p* < 0.001) (Table 2).

**Table 2:** Comparison of delivery mode before and after HSTP.

		Mode of delivery		*p* Value*
		Cesarean section	NVD	
		Frequency (%)	Frequency (%)	
Year	2013 (before HSTP)	3214 (42.6%)	4327 (57.4%)	<0.0001
	2014 (after HSTP)	4125 (38.6%)	6562 (61.4%)	

Abbreviations: HSTP, health system transformation plan; NVD, normal vaginal delivery.

* *χ* ^2^ tests.

The nonparametric *χ* ^2^ test was used to test the difference between the number of NVDs and CS deliveries in mothers of different age groups before and after implementing HSTP. There was a significant difference in the number of NVDs and CS deliveries before and after implementing HSTP in the age groups of 16–20 years (*p* < 0.0001), 21–25 years (*p* < 0.0001), 26–30 years (*p* < 0.04), 31–35 years (*p* < 0.015), and over 40 years (*p* < 0.04). Conversely, there was no significant difference in the age groups under 15 years (*p* < 0.37), or 40–36 years (*p* < 0.053). After implementing the plan, improvements in NVD rates were observed in mothers over 31 years of age (Table 3).

**Table 3:** Comparison of delivery mode in maternal age groups before and after HSTP.

Age group of mothers	Before HSTP						After HSTP						*p* Value
	NVD		CS		Total		NVD		CS		Total		
	Frequency	Percentage	Frequency	Percentage	Frequency	Percentage	Frequency	Percentage	Frequency	Percentage	Frequency	Percentage	
Missing data	28	0.6	4	0.1	32	0.4	11	0.2	8	0.2	19	0.2	‐
<15	21	0.5	6	0.2	27	0.4	26	0.4	4	0.1	30	0.3	0.378
16–20	671	15.5	232	7.2	903	12.0	975	14.9	204	4.9	1179	11.0	<0.0001
21–25	1313	30.3	710	22.1	2023	26.8	1930	29.4	832	20.2	2762	25.8	<0.0001
26–30	1253	29.0	975	30.3	2228	29.5	1891	28.8	1311	31.8	3202	30.0	0.039
31–35	694	16.0	792	24.6	1486	19.7	1156	17.6	1121	27.2	2277	21.3	0.015
36–40	285	6.6	397	12.4	682	9.0	468	7.1	537	13.0	1005	9.4	0.053
>40	62	1.4	98	3.0	160	2.1	105	1.6	108	2.6	213	2.0	0.04

Abbreviations: CS, cesarean section; HSTP, health system transformation plan; NVD, normal vaginal delivery.

**Figure 1:**
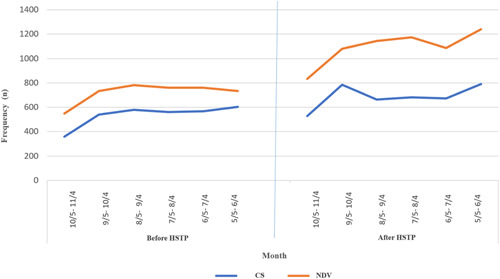
Frequency of deliver in teaching hospitals in the 6 months, from May to November in 2013 and 2014.

According to Figure 1, when comparing the monthly number of NVDs and CS deliveries during the 6 months before and after the HSTP, the rate of deliveries during the first 2 months after the HSTP differed significantly from the corresponding period before the plan. The number of NVDs increased by 2% within 5 months after implementing the plan, that is, from the second half of October to the second half of November in 2014 (Table 4).

**Table 4:** Frequency of deliver in teaching hospitals in the 6 months, from May to November in 2013 and 2014.

Period of time	Before HSTP				After HSTP				*p* Value
	CS		NVD		CS		NVD		
	Frequency	Percentage	Frequency	Percentage	Frequency	Percentage	Frequency	Percentage	
May 5 to June 4	359	11	548	13	527	13	834	13	0.68
June 5 to July 4	540	17	733	17	785	19	1080	16	0.85
July 5 to August 4	578	18	782	18	663	16	1145	17	0.001
August 5 to September 4	560	17	760	18	681	17	1173	18	0.001
September 5 to October 4	568	18	759	18	672	16	1086	17	0.01
October 5 to November 4	602	19	734	17	790	19	1241	19	<0.0001
Missing data	7	0	11	0	7	0	3	0	‐

Abbreviations: CS, cesarean section; HSTP, health system transformation plan; NVD, normal vaginal delivery.

There was no significant difference in the number of single deliveries (codes Z37.0 and Z37.1), twins (codes Z37.2–Z37.4), and triplets (codes Z37.5–Z37.7) before and after implementing HSTP (*α* = 0.05, confidenece interval [CI] = 95%, *p* > 0.8). Two hundred and thirty‐eight deliveries in 2 years were multiple births. The number of multiple births increased by 0.2% in 2014 compared to 2013. Of all 18,228 deliveries, 288 and 229 were dead births in 2014 and 2013, respectively (Table 5).

**Table 5:** Frequency of deliveries based on the code Z37 for the outcome of delivery.

		Before HSTP				After HSTP			
		Cesarean		NVD		Cesarean		NVD	
		Frequency	Percentage	Frequency	Percentage	Frequency	Percentage	Frequency	Percentage
Missing data		2	0.1	5	0.1	2	0.0	4	0.1
Z37.0	Single live birth	3096	96.3	4207	97.2	3959	96.0	6441	98.2
Z37.1	Single stillbirth	23	0.7	106	2.4	42	1.0	95	1.4
Z37.2	Twins, both liveborn	81	2.5	4	0.1	103	2.5	13	0.2
Z37.3	Twins, one liveborn and one stillborn	5	0.2	1	0.0	5	0.1	3	0.0
Z37.4	Twins, both stillborn	1	0.0	1	0.0	4	0.1	3	0.0
Z37.5	Other multiple births, all liveborn	6	0.2	0	0.0	8	0.2	0	0.0
Z37.6	Other multiple births, some liveborn	0	0.0	2	0.0	1	0.0	0	0.0
Z37.7	Other multiple births, all stillborn	0	0.0	1	0.0	1	0.0	3	0.0

Abbreviations: HSTP, health system transformation plan; NVD, normal vaginal delivery.

According to ICD‐10 codes registered in the records of patients in 2013 and 2014, the reason for CS is not specified in the 2778 and 2056 records, respectively. In addition, codes z82.9 and z84.9 have been reported as reasons for CS. Other reasons recorded in the records include multiple births (code O84), breech delivery (code O32.1), elective delivery (code O82.0), previous CS (code O34.2), and other codes (O82.1, O82.2, and O82.8). CS because of unknown reasons was the most prevalent type of CS, followed by previous CS. There was no statistically significant difference in the number of previous CS before and after HSTP implementation (*p* > 0.05).

The highest percentage of previous CS after implementing HSTP was observed in mothers aged 26–30 and 31–35 years. The number of previous CS differed significantly between age groups 26–30, 36–40, and over 40. However, the number of previous CS decreased in the 31–35 age group. In addition, the highest percentage of elective CS was observed in mothers aged 26–30 years. Before the plan, the highest percentage of CS was observed in mothers aged 31–35 years (Table 6).

**Table 6:** Frequency and percentage of previous CS (code O34.2) and elective CS (code O82.0) in mothers of different age groups before and after implementing HSTP.

O34.2	Before HSTP		After HSTP		
	Frequency	Percentage	Frequency	Percentage	*p* Value
Missing data	3	0.4	2	0.2	‐
<15	1	0.1	0	0.0	‐
16–20	23	2.8	23	2.2	1
21–25	131	16.0	164	15.5	0.55
26–30	254	31.0	359	34.0	<0.0001
31–35	262	32.0	298	28.2	0.128
36–40	126	15.4	175	16.6	0.005
>40	20	2.4	35	3.3	0.43
Total	820	100	1066	100	<0.0001

	Before HSTP		After HSTP		
O82.0	Frequency	Percentage	Frequency	Percentage	*p* Value
16–20	5	12.5	0	0.0	‐
21–25	5	12.5	7	25.9	0.56
26–30	10	25.0	9	33.3	0.81
31–35	16	40.0	6	22.2	0.33
36–40	3	7.5	5	18.5	0.48
>40	1	2.5	0	0.0	‐
Total	40	100	27	100	

Abbreviations: CS, cesarean section; HSTP, health system transformation plan.

## 4 DISCUSSION

### 4.1 Principal findings

Promotion of NVD and reduction of CS are the main priorities of the HSTP.^20^ According to codes recorded in patients' records in MUMS teaching hospitals, the CS rate during the past 6 months has declined from 42.6% in 2013 to 38.6% in 2014, indicating a significant difference with a 4% reduction after implementing the first phase of the plan (CI = 99%; *p* < 0.001). The number and percentage of NVDs in MUMS teaching hospitals increased after implementing the plan, implying the HSTP's efficiency in encouraging mothers to undergo NVD. Notably, free services may have contributed to an increased number of NVDs in public hospitals. These findings were consistent with previous research.^1^, ^14^, ^22^, ^23^, ^24^

According to the Iranian MHME, the CS rate in Iran has decreased by 6.5%. These findings indicate much lower rates of CS in Mashhad teaching hospitals than in other hospitals in the country. Since the study has covered teaching hospitals in Mashhad, the impact of the NVD program on promoting NVD is more likely to be lower in big cities than in small towns.

In line with this study, Zaboli et al. reported a 4.75% increase in NVDs after implementing HSTP in Kerman public hospitals, although the difference was not statistically significant.^23^ In another study, Lotfi et al. reported that implementing HSTP leads to a short‐term reduction in CS rates in hospitals affiliated with the Shiraz University of Medical Sciences (SUMS).^25^ The lower reduction in the number of CS in this study may be due to not covering all hospitals in Mashhad, including social security, private, and public nonteaching hospitals. Implementing HSTP has positively affected the number of NVDs and CS deliveries, with a significant difference between the numbers of deliveries 6 months after the plan compared to the previous year. This finding agrees with the results reported by Dehghan et al., who studied teaching and private hospitals in Yazd in 2013 and 2014.^24^

In a study conducted by Rooeintan et al. in Shiraz, the number of vaginal deliveries increased in public hospitals after implementing HSTP. In contrast, after the plan, the number of NVDs decreased while the number of CS deliveries increased in private hospitals. A decrease in the rate of vaginal deliveries in private hospitals was attributed to free services in public hospitals. Similarly, an increase in CS deliveries in these hospitals was attributed to a dearth of midwives who are experts in CS delivery in public hospitals.^1^ On the other hand, this may be because in Iran, with the implementation of the HSTP plan, unlike before, the fee paid to specialists for NVD was higher than for CSs, and after each CS, if there was no specific medical reason, specialists were liable to be questioned.^14^ Jabbari et al. reported a significant difference between the number of NVDs and CS deliveries before and after HSTP. They found that HSTP has significantly diminished CS and increased NVD in public hospitals and increased CS in private hospitals.^6^ This may be because NVD services have become free in teaching hospitals. Women with poor financial conditions and residents of suburbs and villages who previously did not go to hospitals for their deliveries were also encouraged to go to teaching hospitals due to the benefits of free NVD. Moradi et al.^21^ studied the effect of HSTP on promoting NVD rates and found that free‐of‐cost services, encouraging mothers to undergo NVD by obstetrics and gynecology specialists, and providing proper training to choose the type of childbirth have contributed to increasing the rate of NVDs.^21^ Furthermore, Behzadifar et al. reviewed studies on the effect of HSTP on CS in Iran and found a positive effect of HSTP on CS reduction in the country. However, after nearly 4 years of implementing this plan, a 10% annual reduction in CS has not yet been realized. They reported that increased accessibility to childbirth services and community‐based training through mass media could help alter mothers' mindsets about CS in Iran.^14^ Meanwhile, mobile‐based interventions and information systems can be a good option for training and increasing mothers' awareness^26^ due to their ease of use^27^, ^28^ and being available at any time and place.^29^, ^30^

Mathers' demand is an influential factor in increasing CS rates. Therefore, educating and promoting mothers' awareness of CS risks, NVD benefits, and effective, painless delivery methods will efficiently reduce CS rates. ^31^ An MHME's policy to promote vaginal delivery is to promote uncomplicated childbirth, which has significantly contributed to increasing the number of vaginal deliveries.^1^ According to the results, the percentage of NVDs in Mashhad teaching hospitals has been growing since mid‐July 2014, that is, 2 months after implementing HSTP, with a significant difference in the monthly delivery rates compared to the previous year. Six months after implementing the plan in 2014, 4125 CS deliveries have been recorded in Mashhad teaching hospitals. Concerning the maternal age at delivery, the highest percentage (77.1%) of deliveries was among 20‐ to 35‐year‐old mothers. 79.2% of mothers undergoing CS are in this age group. According to the findings of this study, there is a significant difference in previous CS before and after HSTP (*p* < 0.001), but the percentage of previous CS has not altered significantly in 2 years. In 2014, after implementing HSTP, the rate of elective CS was 0.7%, indicating a 0.5% reduction compared to the previous year. However, 33% of elective CS deliveries were done in the age group of 26–30, followed by the age groups of 21–25, 31–35, and 36–40. The results revealed a 13.92% reduction in elective CS deliveries from 2013 (40 cases) to 2014 (27 cases).

According to studies, one of the main reasons for CS is a history of previous CS delivery.^31^, ^32^ In this study, the highest percentage of elective CS was found in the age group of 31–35 years. Concerning the highest delivery rate in this age group, it is essential to encourage women to NVD and appropriately plan to avoid CS childbirth. The number of elective CS cases in records merely recording elective CS cases was 40 in 2013 and 27 in 2014, with the percentage of previous elective CS decreasing from 1.2% to 0.7. The number of elective CS before and after HSTP did not differ significantly (*p* < 0.11); approximately 20% of deliveries were to women in the age group of 31–35. The percentage of elective CS decreased by nearly 50% in this age group, accounting for the largest decrease in elective CS rates. Similarly, there was a significant difference in the number of CS deliveries before and after implementing HSTP among 31–35 years old mothers (*p* < 0.33). The reason for CS was unknown or not mentioned in nearly 65% of records studied. Recording the preferred CS code could assist in researching, managing, and optimally reducing CS rates in the future. These findings were in contrast to the study of Rydahl et al. They reported that CS increased with maternal age in Denmark.^33^

### 4.2 Strengths and limitations

This study epidemiologically investigated deliveries based on the ICD‐10 system. Hence, deliveries were classified based on a coherent and reliable classification system. Also, examining the existing conditions based on an international tool helps to create a unified picture of errors and how to handle them.^34^, ^35^ This work was a multicenter study, thus allowing generalization of the results. However, there were some limitations. First, the study was carried out in MUMS teaching hospitals, and the status of other centers and private hospitals remains unclear. Second, we failed to compare our results with the findings of other countries because of the lack of similar research. Third, the quality of coding was not examined in this study. However, considering that the process of coding medical records in the hospitals under study was done by clinical experts, and then the quality of the codes is examined by HIM experts in the coding department. The authors assumed the high accuracy and adequacy of the assigned codes.

## 5 CONCLUSION

This study was conducted at teaching hospitals in Mashhad, where an increase in the number of NVDs and a decrease in the rate of CS were observed after implementing HSTP. Since previous CS is the most common reason for previous CS, it is pivotal to consider women aged 21–35 years, who have the highest chance of pregnancy. Investigating the effect of the NVD program on promoting NVD in this age group can achieve measurable results. Planning to avoid previous CS and encourage NVD among 21‐ to 35‐year‐old women can help realize HSTP objectives. Concerning the free‐of‐cost services provided with NVD in public centers and the higher rate of CS in private hospitals, it is crucial to conduct similar research in private hospitals and hospitals in other cities to achieve more precise results.

## AUTHOR CONTRIBUTIONS

**Masoumeh Sarbaz**: Conceptualization; project administration; supervision; writing—review and editing. **Seyyedeh Fatemeh Mousavi Baigi**: Formal analysis; methodology; writing—original draft; writing—review and editing. **Fereshte Manouchehri Monazah**: Data curation. **Nooshin Dayani**: Data curation; investigation; writing—review and editing. **Khalil Kimiafar**: Conceptualization; funding acquisition; project administration; supervision; writing—review and editing.

## CONFLICT OF INTEREST STATEMENT

The authors declare no conflict of interest.

## ETHICS STATEMENT

This study was approved by the ethical committee of MUMS (approval number IR.MUMS.REC.1394.609). Privacy and confidentiality were maintained throughout the research period; each record was number coded without any personal identification.

## TRANSPARENCY STATEMENT

The lead author Khalil Kimiafar affirms that this manuscript is an honest, accurate, and transparent account of the study being reported; that no important aspects of the study have been omitted; and that any discrepancies from the study as planned (and, if relevant, registered) have been explained.

## Data Availability

Data available in article supplementary material.
